# Involvement of Lipids in Alzheimer’s Disease Pathology and Potential Therapies

**DOI:** 10.3389/fphys.2020.00598

**Published:** 2020-06-09

**Authors:** Hannah Chew, Victoria A. Solomon, Alfred N. Fonteh

**Affiliations:** ^1^Huntington Medical Research Institutes, Pasadena, CA, United States; ^2^University of California, Los Angeles, Los Angeles, CA, United States; ^3^University of Southern California, Los Angeles, CA, United States

**Keywords:** amyloid precursor protein, apolipoproteins, blood-brain barrier, energy metabolism, inflammation, late-onset Alzheimer’s disease, mitochondria, myelination

## Abstract

Lipids constitute the bulk of the dry mass of the brain and have been associated with healthy function as well as the most common pathological conditions of the brain. Demographic factors, genetics, and lifestyles are the major factors that influence lipid metabolism and are also the key components of lipid disruption in Alzheimer’s disease (AD). Additionally, the most common genetic risk factor of AD, APOE ϵ4 genotype, is involved in lipid transport and metabolism. We propose that lipids are at the center of Alzheimer’s disease pathology based on their involvement in the blood-brain barrier function, amyloid precursor protein (APP) processing, myelination, membrane remodeling, receptor signaling, inflammation, oxidation, and energy balance. Under healthy conditions, lipid homeostasis bestows a balanced cellular environment that enables the proper functioning of brain cells. However, under pathological conditions, dyshomeostasis of brain lipid composition can result in disturbed BBB, abnormal processing of APP, dysfunction in endocytosis/exocytosis/autophagocytosis, altered myelination, disturbed signaling, unbalanced energy metabolism, and enhanced inflammation. These lipid disturbances may contribute to abnormalities in brain function that are the hallmark of AD. The wide variance of lipid disturbances associated with brain function suggest that AD pathology may present as a complex interaction between several metabolic pathways that are augmented by risk factors such as age, genetics, and lifestyles. Herewith, we examine factors that influence brain lipid composition, review the association of lipids with all known facets of AD pathology, and offer pointers for potential therapies that target lipid pathways.

## Background

### The Importance of Cellular Lipid Membranes

**FIGURE 1:**
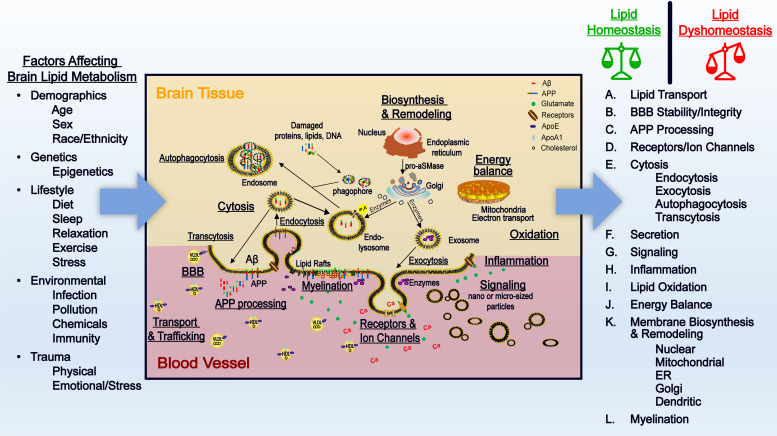
Factorsthat affect brain lipid metabolism and the importance of lipids in healthy aging and AD. *Factors that affect brain lipid metabolism* – Demographic factors, genetics, lifestyle, the environment, and trauma can influence lipid metabolism in the brain. Interestingly, these factors that influence lipid metabolism are also recognized risk factors of AD. Abnormalities in lipid metabolism can contribute to dysfunctional brain networks that associate with AD pathology. *Importance of lipid metabolism in brain function and AD pathology* – In healthy aging, normal transport of lipids through apolipoproteins contribute to the function of the brain. Homeostatic control of the brain lipid environment is responsible for sustaining a normal BBB, providing the right environment for normal APP processing, the right composition for ion channels and receptors, cytosis, vesicle formation, and secretion, signaling, inflammation, oxidation, energy balance, and membrane biosynthesis and remodeling. Dyshomeostasis in lipid delivery into the brain and its metabolism attributes to disturbed BBB, abnormal APP processing, disturbance in cytosis, signaling, energy balance, and enhanced/sustained inflammation and oxidation. Over time, these processes lead to neuronal death that is the hallmark of AD pathology.

Cell membranes are composed of several lipid classes and membrane-bound proteins/receptors that interface cellular organelles, and cells with their environment. It is now recognized that these membrane lipids are important in maintaining cellular functions. Several studies show that perturbation of membrane lipids can have devastating consequences on the brain. These changes underlie Alzheimer’s disease (AD) pathology depicted in Figure 1. We will examine factors that affect lipid metabolism, describe the functions of brain lipids, and examine the consequences and contributions of lipid dyshomeostasis on AD pathology.

### Brain Lipids in Healthy Aging and AD Pathology

Most of the brain is composed of lipids, which can be grouped as sphingolipids, glycerophospholipids, and cholesterol (Svennerholm et al., 1994; O’Brien and Sampson, 1965; Kishimoto et al., 1969). The brain consists of straight-chain monocarboxylic acids ranging from C_12_ to C_26_, and omega-3 (n-3) and omega-6 (n-6) fatty acids are most abundant (Kishimoto et al., 1969; Siegel, 1999). Docosahexaenoic acid (DHA) and eicosapentaenoic acid (EPA) are prominent polyunsaturated fatty acids (PUFA) in the brain that are derived from alpha-linolenic acid (ALA), an omega-3 fatty acid (Chappus-McCendie et al., 2019). Arachidonic acid (AA) and docosatetraenoic acid (DPA) constitute a large proportion of PUFA’s that are derived from linolenic acid (LNA), an omega 6 fatty acid (Leonard et al., 2000; Sinclair et al., 2007).

## Factors That Affect Brain Lipids

### Demographic Factors That Influence Brain Lipids

#### Brain Lipids Changes in Aging

These PUFA’s are incorporated into membrane phospholipids and therefore play a significant role in structural integrity and function of cell membranes. Lipid metabolism is changed during aging (Montanini et al., 1983; Yehuda et al., 2002; Whelan, 2008; Denis et al., 2015; Cutuli, 2017; Chappus-McCendie et al., 2019), as shown by a decline in omega-3 fatty acids and an increase in lipid peroxidation (Chen et al., 2017). Omega-3 fatty acids have antioxidant properties, and a lack of these fatty acids in one’s diet may accelerate neuronal degeneration (Yehuda et al., 2002; Janssen and Kiliaan, 2014). Susceptibility of lipids to peroxidation increases with age (Bourre, 1991; Spiteller, 2010; Denis et al., 2015; Chen et al., 2017), which supports using the level of oxidative stress as a critical determinant of neuronal health and longevity (Hulbert et al., 2006). Previous studies have suggested that DHA and EPA may protect against peroxidation and the effects of age-related brain pathology (Hasadsri et al., 2013; Chen et al., 2017). Lipids are involved in cellular signaling, energy balance, blood-brain barrier (BBB), and inflammation (Song et al., 2008; Willis et al., 2009), and such age-dependent lipidome changes that disrupt these functions may contribute to neurodegenerative diseases (Arnoldussen et al., 2016; Bos et al., 2016; Hooper et al., 2018; Luo et al., 2018; McNamara et al., 2018; Lepping et al., 2019), such as Alzheimer’s disease (AD) (Schmitt et al., 2014; Hussain et al., 2019).

#### Lipids and Race/Ethnicity

Race and ethnicity play a significant role in the risk of AD and related disorders. In 2014, nearly 5 million people over the age of 65 had been diagnosed with Alzheimer’s disease or related dementias (ADRD) (Matthews et al., 2019). African Americans and Hispanics had the highest prevalence of ADRD (13.8% and 12.2%, respectively), while ADRD was least common in Asian and Pacific Islanders (8.4%), followed by American Indian/Alaska Natives (9.1%), non-Hispanic whites (10.3%), and people with two or more races (11.5%) (Matthews et al., 2019). Ethnic and racial backgrounds impact many aspects of health, including diet, stress, access to medical treatment, and biological factors of disease. From past research, we can clearly see the ways in which ethnicity, race, and lipids overlap. Most clearly seen in the high incidence of both dyslipidemia, or abnormal amounts of lipids in the blood, and cardiovascular disease observed in minority populations (Frank et al., 2014), race/ethnic disparities affect the regulation of lipid metabolism. Increased concentrations of triglycerides (TG) and decreased levels of lipid carriers, such as HDL-C (high-density lipoprotein-cholesterol) in the blood of Mexican, Filipino, Indian, and Vietnamese people compared to whites may provide a possible explanation for higher risk of both ADRD and cardiovascular disease within these populations (Frank et al., 2014; Gazzola et al., 2017). HDL-C is often referred to as “good cholesterol,” has beneficial antioxidant and anti-inflammatory effects in the body, and has been observed to modulate ß-amyloid (Aß) production in the brain, a key biomarker of AD pathogenesis (Reitz, 2012; Hottman et al., 2014). Lowered levels of HDL-C have been associated with increased cognitive decline and poor cardiovascular health outcomes (Hottman et al., 2014). TG, which is increased in almost every minority population, except African Americans, has been shown to relate to central leptin- and insulin resistance in the brain and decreases in cognition (Sumner, 2009; Frank et al., 2014; Banks et al., 2018). In light of the less marked changes in lipid make-up and metabolism seen in African American populations at increased risk of ADRD, it has been suggested that African Americans are underdiagnosed with metabolic syndromes and vascular-cognitive disorders (Sumner, 2009). Furthermore, it has been observed that there is a differential expression of various molecular biomarkers of AD (phosphorylated tau and total tau) in African Americans compared to whites (Morris et al., 2019), suggesting even small, imperceptible changes in lipid distribution in this population may be sufficient to affect cognition negatively. It is important to note that despite the disproportionate impact ADRD has on minority populations, these individuals continue to be considerably underrepresented in ADRD research, contributing to large gaps in our understanding of brain lipid metabolism as it pertains to race and ethnicity (Gilmore-Bykovskyi et al., 2019).

#### Lipids and Sex

Sex continues to be one of the largest risk factors for developing AD. Females not only makeup two-thirds of all cases of AD diagnoses but also possess a greater lifetime risk of dementia compared to men due to longer life expectancy (Viña and Lloret, 2010; Mielke, 2018). Increased prevalence and risk of AD and other age-related disorders among females have been attributed to not only extended life expectancy but also to sudden decreases in estrogen post-menopause, among many other factors including education level and mental health status (Viña and Lloret, 2010; Mielke, 2018). Despite the many factors that may contribute to increased risk of AD in women, the contribution of sex-hormone levels and differential lipid distribution play evident roles in cognitive decline are not fully understood. Not only is fat in the form of TG distributed differently in the adipose tissue of male and females, which can be attributed in part to sex-hormone signaling, but concentrations of long-chain PUFAs (LC-PUFAs) have also been observed to be increased in women pre-menopause compared to men (Decsi and Kennedy, 2011; Lohner et al., 2013). Correspondingly, a positive association has been established between omega-3 LC-PUFA biosynthesis, i.e., the production of EPA and DHA, and circulating concentrations of estrogen and progesterone (Childs et al., 2008). Estrogen, an ovarian steroid hormone, is hypothesized to affect lipid metabolism at several points during biosynthesis, including playing a key role in lipid transport and exchange, increasing expression of metabolic enzymes, and reducing the oxidation of α-linoleic acid (ALA), the deriving fatty acid in n-3 LC-PUFA production (Childs et al., 2008; Decsi and Kennedy, 2011; Lohner et al., 2013; Palmisano et al., 2018). Estrogen has also been directly associated with inhibiting memory function impairment in premenopausal women following the surgical removal of their ovaries and loss of the ability to produce estrogen endogenously (Duka et al., 2000; Sherwin, 2012). In a study of *trans*-sexual subjects, those transitioning from male to female and receiving estrogen observed an increase in DHA plasma levels while those transitioning from female to male and receiving testosterone treatment experienced a marked decrease in plasma DHA (Giltay et al., 2004). The decrease in estrogen levels, as seen in post-menopausal women, has also been associated with increased TG content and lower HDL-C, both of which have been linked to cognitive decline (Derby et al., 2009; Anceline et al., 2014). This is to say, the increased prevalence and risk for AD among women can be explained in part by the abrupt decrease in estrogen production that accompanies the post-menopausal state. Not only does the lack of estrogen decrease concentrations of anti-inflammatory LC-PUFAs and HDL-C in the body, but it also increases TG levels, augmenting secretion of VLDL (very-low density lipoprotein), a lipid carrier known to induce neuroinflammation (Burgess et al., 2006; Chen et al., 2014; Nägga et al., 2018). Additionally, genetic factors, such as ApoE status, and social determinants, such as education, mental illness, and diet, interact with the post-menopausal state to amplify these detrimental effects, increasing risk of AD.

### Lipids and Lifestyle

#### Diet

Dietary lipids play an integral part in physiological lipid metabolism and, consequently, in the risk of AD and cardiovascular disease. Essential fatty acids like DHA (n-3) and AA (n-6) are largely derived from the dietary consumption of their shorter-chained, slightly less-saturated counterparts ALA (n-3) and LA (n-6), respectively (Schmitz, 2008; Morris and Tangney, 2014). After consumption of these deriving fatty acids, the body is able to anabolize them, creating the LC-PUFAs that contribute to neural processes (Morris and Tangney, 2014). Early on in human existence, our diet consisted of an equal balance of n-6 to n-3 essential fatty acids, but as we have evolved, the n-3 to the n-6 ratio of dietary fatty acids has greatly shifted to one side (Simopoulos, 2006). Today, the Western diet has a ratio of about 17 to 1 n-6 to n-3 fatty acids, meaning most Americans have a lot more LA, AA, and DPA in their bodies, which are able to produce relatively large quantities of inflammatory and oxidative mediators (Simopoulos, 2006). Increased ratios of n-6 to n-3 dietary fatty acids have also been directly associated with increased cognitive decline and risk of AD (Loef and Walach, 2013; MacDonald-Wicks et al., 2019). DHA, on the other hand, an n-3 LC-PUFA usually found in fish and algae, is not largely found in the Western diet. Studies suggest, however, that DHA supplementation may work to combat neuroinflammation, oxidative stress, and cognitive decline. Fish oil supplements containing large amounts of DHA, given to older adults with varying levels of cognition, found that supplementation resulted in decreased brain atrophy and less cognitive decline compared to controls in an *APOE* allele-dependent manner (Daiello et al., 2015). Similarly, Morris et al. observed among subjects over the age of 65 that those who ate fish at least once a week had 60% less risk of AD than those who rarely or never ate fish (Morris et al., 2003). Dietary DHA has also been shown to improve cognition, memory, and brain development from the earliest stages of life through adulthood (Dunstan et al., 2008; McNamara et al., 2010; Muldoonm et al., 2010; Stonehouse et al., 2013; Weiser et al., 2016).

It is important to note that diet can be particularly impacted by race/ethnicity, as well as physical geography, helping to explain differences in AD risk among ethnic groups. According to a global survey of 298 studies, highest levels of DHA and EPA, another n-3 fatty acid, were observed among Japanese, Scandanavian, and indigenous populations, as well as in areas where the Westernized diet had not been fully adopted (Stark et al., 2016). Authors of this survey argue increased consumption of seafood, as dictated by culture or geographical location, greatly impact n-3 LC-PUFA levels in the bloodstream, which offer protective cognitive effects at every stage in life (Joffre et al., 2014; Stark et al., 2016; Weiser et al., 2016).

### Genetical Evidence for the Importance of Lipid Metabolism in AD Pathology

#### Genetic Risk Factors of AD-Related to Lipid Metabolism

Genome-wide Association Studies GWAS and Transcriptome-Wide Association Studies (TWAS) associate AD pathology with several lipid genes (Shi et al., 2010; Hao et al., 2018). While the APOE4 allele carries the greatest risk for AD, other genes and gene-products commonly associated with AD pathology are linked to or interact with lipid metabolism. Several lipid genes associated with AD pathology have recently been reviewed (Tindale et al., 2017). Table 1 is the list of the major genes from GWAS that are linked with lipid metabolism (Jones et al., 2010).

**TABLE 1:** Lipid metabolism-associated genes with SNP (<0.001) linked with AD from GWAS.

Gene Symbol [*Chromosome location* (*Mb*)] (Jones et al., 2010)	The function of the gene product^#^	Changes and known effects on AD pathology
APOE [*19* (*50*)]	As part of lipoproteins, ApoE is involved in the transport and distribution of lipids into various tissues via plasma and other interstitial fluids (Huang and Mahley, 2014)	Polymorphism of APOE is associated with age of onset (do Couto et al., 1998; Vermunt et al., 2019), cognitive and memory decline (Asada et al., 1996; El Haj et al., 2016), amyloid load (Mecca et al., 2018), cholesterol homeostasis (Leduc et al., 2010), inflammation (Tzioras et al., 2019)
APOC1 [*19* (*50*)]	Involved in HDL and VLDL metabolism, inhibitor cholesteryl ester transfer protein in plasma	Gene polymorphism (Prendecki et al., 2018), oxidative stress (Prendecki et al., 2018), interaction with ApoE (Lucatelli et al., 2011), cognitive impairment (Zhou et al., 2014)
CLU [*8* (*28*)]	Clusterin (ApoJ) is a component of lipoproteins associated with lipids in plasma and CSF	Polymorphism (Shuai et al., 2015; Zhu et al., 2018), interaction with PICALM (Harold et al., 2009; Kamboh et al., 2012), hippocampal function (Erk et al., 2011).
APOC2 [*19* (*50*)]	A component of triglyceride (TG)-rich lipoproteins, including VLDL, HDL), and chylomicrons involved metabolism of these particles; promote VLDL1 secretion, inhibit lipoprotein lipase enzyme activity	Polymorphism associated with AD (Sun et al., 2005), decreased expression associated with increased risk (Lin et al., 2015)
APOC4 [*19* (*50*)]	A lipid-binding lipoprotein thought to play a role in lipid metabolism	Decreased expression associated with increased risk (Lin et al., 2015)
ABCA7 [*19* (*1*)]	Member of the ATP-binding cassette (ABC) superfamily of transporters; catalyzes the translocation of specific phospholipids from the cytoplasmic to the extracellular/lumenal leaflet of the membrane coupled with ATP hydrolysis, lipid homeostasis, binds APOA1, apolipoprotein-mediated phospholipid efflux from cells, cholesterol efflux, lipid raft organization	Polymorphism correlate with memory impairment (Chang et al., 2019), amyloid plaque burden (Zhao Q. F. et al., 2015), cognitive impairment (Chung et al., 2014; Berg et al., 2019)
ABCA1 [*9* (*107*)]	A membrane of the superfamily of ATP-binding cassette (ABC) transporters with cholesterol as its substrate, it functions as a cholesterol efflux pump in the cellular lipid removal pathway	Polymorphism in AD (Chu et al., 2007; Wavrant-De Vrieze et al., 2007; Wang et al., 2013), modulates cholesterol efflux (Shibata et al., 2006; Khalil et al., 2012; Marchi et al., 2019), influences age of onset (Wollmer et al., 2003a)
ABCA12 [*2* (*216*)]	A membrane of the superfamily of ATP-binding cassette (ABC) transporters involved in the transport of molecules across the cellular membrane	SNP with *p* < 0.001 and significantly enriched in AD (Jones et al., 2010)
LIPC [*15* (*57*)]	Hepatic triglyceride lipase is a triglyceride hydrolase and ligand/bridging factor for receptor-mediated lipoprotein uptake	Gene variant might influence AD susceptibility (Xiao et al., 2012)
ATP8A1 [*4* (*42*)]	ATPase Phospholipid Transporting 8A1 catalyzes ATP hydrolysis that is coupled to the transport of aminophospholipids from the outer to the inner leaflet of membranes to maintain their asymmetric distribution	SNP with *p* < 0.001 and significantly enriched in AD (Jones et al., 2010)
ATP8B4 [*15* (*48*)]	Amninophospholipid transport across cell membranes	SNP with *p* < 0.001 and significantly enriched in AD (Jones et al., 2010)
MALL [*2* (*110*)]	Member of the MAL proteolipid family localizes in glycolipid- and cholesterol-enriched membrane (GEM) rafts, and interacts with caveolin-1	SNP with *p* < 0.001 and significantly enriched in AD (Jones et al., 2010)
ATP8A2 [*13* (*25*)]	Involved in flipping phospholipids from the exoplasmic leaflet to the cytosolic leaflet of the cell membrane to generate or maintain membrane lipid asymmetry	SNP with *p* < 0.001 and significantly enriched in AD (Jones et al., 2010)
OSBPL7 [*17* (*43*)] OSBPL9 [*1* (*59*)]	Oxysterol-binding protein (OSBP) family, intracellular lipid receptors Oxysterol-binding protein (OSBP) family, a group of intracellular lipid receptors; cholesterol transfer protein and regulation of Golgi structure and function	Differential expression (Brown III, Theisler et al., 2004; Herold et al., 2016)
SCARB1 [*12* (*124*)]	Scavenger Receptor Class B Member 1 is a plasma membrane receptor for HDL that also mediates cholesterol transfer to or from HDL	Cholesterol efflux and anti-inflammation (Khalil et al., 2012), endocytosis, transcytosis and Abeta removal (Mackic et al., 1998; Srivastava and Jain, 2002)
VPS4B [*18* (*59*)]	Vacuolar Protein Sorting 4 Homolog B involved in late endosomal multivesicular bodies (MVB) pathway. Degradation of lysosomal enzymes and lipids.	SNP with *p* < 0.001 and significantly enriched in AD (Jones et al., 2010)
ABCG1 [*21* (*42*)]	Coupled to ATP hydrolysis, catalyzes the efflux of sphingomyelin, cholesterol, and oxygenated derivatives like 7-beta-hydroxycholesterol.	Cholesterol efflux (Hirsch-Reinshagen and Wellington, 2007; Wollmer et al., 2007; Marchi et al., 2019)
LIPG [*18* (*43*)]	Diverse class of lipase enzymes includes diacylglycerol lipase (DAGL) and lipoprotein lipase (LPL) and endothelial lipase (LIPG). Hydrolyzes HDL more efficiently than other lipoproteins	Polymorphism and mutation (Baum et al., 1999; Blain et al., 2006), cholesterol homeostasis (Blain and Poirier, 2004; Fidani et al., 2004), stimulation in nucleus basalis and hippocampus (Farooqui et al., 1988)
PCTP [*17* (*51*)]	Phosphatidylcholine (PC) Transfer Protein; PC synthesis and metabolism, binds single PC molecule and transfers between membranes	Cholesterol transport (Knebl et al., 1994; Demeester et al., 2000)
SLC27A4 [*9* (*130*)]	Family of fatty acid transport proteins; translocation of long-chain fatty acids across the plasma membrane, has acyl-CoA ligase activity for long-chain and very-long-chain fatty acids (VLCFAs)	SNP with *p* < 0.001 and significantly enriched in AD (Jones et al., 2010)
NPC1 [*18* (*19*)]	Intracellular cholesterol transporter which is important in cholesterol removal from endosomal/lysosomal compartment	Increase expression (Kagedal et al., 2010; Maulik et al., 2015), neurocognitive deficit (Johnen et al., 2018)
APOA1 [*11* (*116*)]	Apolipoprotein A-I is the major protein HDL in plasma. It promotes cholesterol efflux from tissues to the liver for excretion and is a cofactor for lecithin cholesterol acyltransferase (LCAT), an enzyme that forms cholesteryl ester	Polymorphism and decreased expression (Helbecque et al., 2008; Smach et al., 2011; Shibata et al., 2013; Lin et al., 2015)
APOC3 [*11* (*116*)]	A component of triglyceride-rich VLDL, and HDL in plasma. Important in triglyceride homeostasis: promotes hepatic VLDL1 assembly and secretion, attenuates hydrolysis and clearance of triglyceride-rich lipoproteins, impairs TRL lipolysis by inhibiting lipoprotein lipase and the hepatic uptake of TRLs by remnant receptors	Polymorphism and decreased expression (Lin et al., 2015; Sun et al., 2005)
APOA4 *[11 (116*)]	Apolipoprotein A4 is a major component of HDL and chylomicrons. Important in chylomicrons and VLDL secretion and catabolism. Required for lipoprotein lipase activation by ApoC-II, a potent activator of LCAT	Decreased expression (Lin et al., 2015), enhanced susceptibility (Papassotiropoulos et al., 2005)
AGTR1 [*3* (*1490*)]	Angiotensin II is a primary regulator of aldosterone secretion	Signal transduction abnormality (Pang et al., 2019)
SOAT1 [*1* (*177*)]	Sterol O-Acyltransferase-1 is an acyltransferase that catalyzes the formation of fatty acid and cholesterol esters, which is important in lipoprotein assembly and dietary cholesterol absorption. It may also act as a ligase	Polymorphism (Lamsa et al., 2007), Cholesterol levels, amyloid load (Wollmer et al., 2003b)

*^#^https://www.genecards.org/.*

Genome-wide Association Studies suggest that age-related changes in brain lipid metabolism may be essential to healthy aging and longevity (Tindale et al., 2017). Identification of AD-related genes and how these interact with specific risk factors may provide the rationale for designing effective therapies.

The onset of age related disease can be accelerated with suppression of anti-aging genes, such as Sirtuin 1 (SIRT1). SIRT1 is a histone deacetylase involved with gene expression related to metabolic activity (Grabowska et al., 2017). SIRT1 interacts with lipid metabolism regulation and hepatic oxidative stress and inflammation (Ding et al., 2017). It also regulates circadian rhythms in the liver and brain, maintaining the body’s regulation of glucogenesis, fatty acid beta-oxidation, and cholesterol biosynthesis (Bellet et al., 2016). Its involvement in metabolism explains its effects on energy metabolism, neurogenesis, glucose and cholesterol metabolism, and amyloidosis. Sirt 1 also contributes to neuron apoptosis and survival. Downregulation of this anti-aging gene may lead to acceleration of neurodegenerative disease. Nutritional interventions, such as a reduction in overconsumption of carbohydrates, are recommended because they may be associated with preventing cell senescence and maintaining anti-aging gene activity (Martins et al., 2017). SIRT1 expression promotes APP processing on a non-amyloidogenic pathway and clearance of tau from the brain (Herskovits and Guarente, 2014). SIRT1’s deacetylase activity increases the activity of lysosome-related genes, facilitating Aβ degradation (Li et al., 2018). SIRT1 is a potential therapeutic target for AD because of its involvement in many amyloid beta and cholesterol pathways.

## Contribution of Lipids to AD Pathology

Although the brain has a very high concentration of long-chain omega-3 and omega-6 fatty acids, there is no conclusive explanation for how these fatty acids participate in various signaling cascades and in AD (Torres et al., 2014; Mohaibes et al., 2017). However, lipodomic studies related to AD pathology have demonstrated a decrease in DHA levels within the brain, predominantly in the hippocampus (Belkouch et al., 2016). Damage to the hippocampus is associated with impaired learning and memory abilities, a symptom of AD onset (Sarrafpour et al., 2019). With growing evidence that AD is associated with dysregulation of fatty acid metabolism, fatty acid levels may be potential biomarkers of this disease (Fonteh et al., 2014; Wong et al., 2017). In addition to omega fatty acids, the levels of several lipids change with AD pathology (Table 2).

**TABLE 2:** Summary of lipids that change in AD.

Lipids	Changes observed in AD
**Fatty acids**	
Omega-3 fatty acids (^#^DHA, EPA, DPA, ALA)	DHA decreased in brains, circulation, and CSF of AD individuals (Fonteh et al., 2014, 2020; de Wilde et al., 2017; Snowden et al., 2017; Hosseini et al., 2020). EPA decreased in brain and circulation of AD individuals (Hosseini et al., 2020). DPA increased in livers of AD (Dyall, 2015). ALA increased in plasma and peripheral tissues (Cherubini et al., 2007).
Omega-6 fatty acids (^#^AA, LA)	AA increased in brains, erythrocytes, and CSF of AD individuals (Thomas et al., 2016; Goozee et al., 2017; Fonteh et al., 2020). LA decreased in AD brain and plasma (Snowden et al., 2017; Cunnane et al., 2012).
Saturated fatty acids (^#^PA, SA, C15:0)	Increased in the CSF and brains of AD individuals (Fonteh et al., 2014). Odd chain saturated fatty acids derived from microbiome or measures of dairy consumption decreased in CSF of AD (Fonteh et al., 2014; Nasaruddin et al., 2016).
Eicosanoids	Pro-inflammatory eicosanoid pathways are upregulated in AD individuals, while anti-inflammatory eicosanoids are decreased (Biringer, 2019). Prostaglandin and thromboxane B_2_ increased in AD brains (Iwamoto et al., 1989). Pro-resolvin mediators, such as lipoxins, are reduced in AD brains (Wang et al., 2015b).
Endocannabinoids	Decreased levels of endocannabinoids and receptors in AD brains (Bedse et al., 2015).
**Glycerolipids**	
Triglycerides	Total TG lipid levels decreased in the serum of individuals with probable AD (Lepara et al., 2009). Polyunsaturated TG decreased in AD brains (Bernath et al., 2019).
**Glycerophospholipids**	
Phosphatidylcholine (PC) Phosphatidylethanolamine (PE) Phosphatidylserine (PS)	Total PC lipids decreased in AD brains (Wood, 2012). PC species decreased in CSF of AD individuals **^#^***PC32:0, PC34p:0/34e:1, PC34:1, PC34:0, PC36:1, PC38a:5 (PC-EPA), PC36:0/38p:6, 38a:6 (PC-DHA)* (Fonteh et al., 2013). PC species decreased in plasma of AD individuals. *PC36:5 (PC-EPA), PC38:6 (PC-DHA), PC40:6 (PC-DHA)* (Whiley et al., 2014). PC species increased in the prefrontal cortex of AD individuals. *PC38:6 (PC-DHA)*, *PC40:6 (PC-DHA)* (Igarashi et al., 2011). Total PE lipids decreased in the hippocampus of AD individuals (Prasad et al., 1998). PE species decreased in the hippocampus of AD individuals. *PE-SA*, *PE-OA*, *PE-AA*, *PE-DHA* (Guan et al., 1999). A decrease in PE plasmalogen in AD (Farooqui and Horrocks, 1998). Total PS lipids decreased in the occipital lobe and inferior parietal lobule of AD brains (Farooqui and Horrocks, 1998).
**Sphingolipids**	
Sphingomyelin (SM) Ceramides (CM) Sulfatides Gangliosides	Total SM lipids lower in CSF of AD individuals (Fonteh et al., 2015). SM species decreased in the CSF of AD individuals **^#^***SM18/14:0, SM18/16:0, SM18/16:1, SM18/17:0* SM species *(SM18/18:0, SM18/18:1)* increased in the CSF of prodromal AD individuals (Kosicek et al., 2012). Total CM lipids increased in AD brains (Filippov et al., 2012). CM species increased in AD brains and plasma *CM16:0 (PA), CM18:0 (SA), CM20:0, CM24:0, CM24:1* (Kim et al., 2017). Total sulfatide levels significantly lower in AD brains in both gray and white matter. The compositional distribution of sulfatide subtypes is unaltered (Han et al., 2002). Ganglioside lipid levels reduced in the temporal lobe of AD brains (Molander-Melin et al., 2005).
**Sterols**	
Cholesterol Oxysterols Hormones	Cholesterol decreased, and oxysterol/cholesterol precursors increased in MCI and sporadic AD brains (Hascalovici et al., 2009). Total oxidized cholesterol increased in AD brains (Heverin et al., 2004). Oxidized cholesterol species decreased in AD brains, *24S-hydroxycholesterol* (Heverin et al., 2004). Oxidized cholesterol species increased in AD brains, *27-hydroxycholesterol* (Heverin et al., 2004). Lower estrogen increases the risk of AD (Ratnakumar et al., 2019; Uddin et al., 2020). Increased basal cortisol levels in the plasma of demented individuals (Csernansky et al., 2006). Association of cortisol with Aβ deposition (Toledo et al., 2012) and with hypometabolism (Wirth et al., 2019).

*^#^DHA, Docosahexaenoic acid (C22:6, n-3); EPA, Eicosapentaenoic acid (C20:5, n-3); DPA, Docosapentaenoic acid (C22:5, n-3); ALA, Alpha-linolenic acid (C18:3, n-3); AA, Arachidonic acid (C20:4, n-6); LA, Linoleic acid (C18:2, n-6); PA, Palmitic acid (C16:0); SA, Stearic acid (C18:0); OA, Oleic Acid (C18:1, n-9); PC, phosphatidylcholine; PCxxa:#, PC specie with xx carbon number and acyl-linked fatty acid at the sn-1 position containing # (number) double bonds; PC**xx**p:**#**, PC specie with xx carbon number and alk-1-enyl (plasmalogen)-linked fatty acid at the sn-1 position containing # (number) double bonds PC**xx**e:**#** PC specie with xx carbon number and alkyl (ether)-linked fatty acid at the sn-1 position containing # (number) double bond; PE, phosphatidylethanolamine; PS, phosphatidylserine; SM, sphingomyelin; CM, ceramide; TG, triglyceride.*

### Lipid Transport: Apolipoproteins

#### Brain Lipoproteins and Their Function

Lipoproteins are molecules with a hydrophobic lipid core composed of cholesterol, esters, and triglycerides and a hydrophilic exterior of phospholipids, apolipoproteins, and free cholesterol (Alaupovic, 1996; Hoofnagle and Heinecke, 2009; Braun and Hantke, 2019). Lipoproteins assist with the transport of lipids and amphipathic compounds throughout the body (Feingold and Grunfeld, 2000). However, circulating plasma lipoproteins differ from those within the CNS because only high-density lipoproteins (HDL) can cross the blood-brain barrier (Balazs et al., 2004). The most abundant apolipoproteins, apolipoprotein E (ApoE), and apolipoprotein J (ApoJ) are synthesized by astrocytes and serve as enzyme cofactors and receptor ligands on HDL (Pitas et al., 1987; Feingold and Grunfeld, 2000; Ito et al., 2014).

Apolipoproteins are greatly involved in metabolism, serving as both activators and inhibitors of metabolic enzymes, ligands for lipoprotein receptors, and providing structural support (Feingold and Grunfeld, 2000; Bolanos-Garcia and Miguel, 2003; Filou et al., 2016). They also regulate lipid transport by controlling interactions with receptors, enzymes, and lipid-transport proteins (Bolanos-Garcia and Miguel, 2003; Ramasamy, 2014). Apolipoproteins have receptor binding domains containing low-density lipoprotein (LDL) receptors that direct lipid and substrate delivery to specific brain cells (Clavey et al., 1995; Dehouck et al., 1997; Herz, 2001). Their amphipathic-helices facilitate lipid-binding and lipid transport (Clavey et al., 1995; Prevost and Kocher, 1999; Elliott et al., 2010). LDL receptors also facilitate the clearance of amyloid peptides through the BBB (Shibata et al., 2000).

#### Contribution of Lipoproteins to AD Pathology

Brain lipoproteins with ApoE are responsible for phospholipid and cholesterol transport (Growdon and Hyman, 2014; Wong et al., 2019). ApoE is mainly expressed in astrocytes and microglia and appears as three major isoforms, ApoE2, ApoE3, and ApoE4, of which ApoE4 is the strongest genetic risk factor for AD (Stone et al., 1997; Ito et al., 2005; Vance and Hayashi, 2010; Chung et al., 2016; Liu et al., 2017; Montoliu-Gaya et al., 2018; Tulloch et al., 2018). ApoE4 demonstrates a lower affinity for lipids than ApoE2 and ApoE3, limiting CNS transport of lipids needed for neuronal remodeling and repair (Bradley and Gianturco, 1986; Barbagallo et al., 1998; Li et al., 2002; Frieden et al., 2017). Furthermore, levels of ApoE LDL receptors directly correlate with Aβ clearance, and promoting the expression of these receptors are potential therapeutic targets for AD treatment (Zhao et al., 2018). ApoJ, also known as clusterin, is expressed in astrocytes, neurons, and ependymal cells (Nuutinen et al., 2005, 2007). This neuroprotectant initiates a defense response to neuronal damage and clears Aβ across the BBB via LDLR-2 (Merino-Zamorano et al., 2016; Nelson et al., 2017; Zandl-Lang et al., 2018). ApoJ’s role in Aβ accumulation and toxicity is still undetermined because variability under different contexts and environments confound results (Foster et al., 2019).

### Lipids and the Blood-Brain Barrier

#### The Blood-Brain Barrier

The blood-brain barrier (BBB) is a semipermeable membrane that carefully regulates the exchange of solutes between blood and brain to maintain CNS homeostasis and block entry of toxins and pathogens into the CNS (Bradbury, 1984; Abbott et al., 2010; Betsholtz, 2014; Daneman and Prat, 2015; Ferreira, 2019; Moura et al., 2019). The integrity of the BBB is largely dependent on its tight junctions (Brown and Davis, 2002; Castro Dias et al., 2019), adherens junction proteins, and ability to control the vesicular movement of macromolecules through transcytosis and pinocytosis (Dehouck et al., 1997; Baldo et al., 2014). The BBB permits free diffusion of gases, such as oxygen and carbon dioxide, but small solutes such as lipophilic molecules and ions enter through receptor-mediated transcytosis or via channels (Fishman et al., 1987; Zlokovic, 2008; Preston et al., 2014; Andreone et al., 2017; Villasenor et al., 2017; Ayloo and Gu, 2019). The BBB is critical in linking multiple major organ systems, and any dysfunction in the lipid bilayer’s ability to act as a barrier may lead to neuronal degeneration (Zhao Z. et al., 2015; Halliday et al., 2016; Muszynski et al., 2017; Nation et al., 2019).

#### Importance of Lipids in BBB Function

In addition to composing the BBB lipid bilayer, lipids, including phospholipids, sphingolipids, and cholesterol, also compose the plasma membrane of vesicles involved with receptor-mediated transcytosis within the CNS (Kramer et al., 2002; Dodelet-Devillers et al., 2009; Campbell et al., 2014; Andreone et al., 2017). The formation and function of vesicles required to transport essential macromolecules across the BBB may be affected by the plasma membrane lipid composition (Lingwood et al., 2009; Lingwood and Simons, 2010; Kaiser et al., 2011). In particular, DHA disrupts the membrane domains necessary to form such transport vesicles and therefore contributes to BBB integrity and suppression of transcytosis (Ouellet et al., 2009; Freund Levi et al., 2014; Pan et al., 2015, 2016; Belayev et al., 2018). There is also recent evidence that the membrane transport protein, Mfsd2a, controls lipid exchange and plays a key role in the transport of DHA into the brain, though this pathway is largely undetermined (Segi-Nishida, 2014; Zhao and Zlokovic, 2014; Keaney and Campbell, 2015; Andreone et al., 2017). Loss of Msfd2a transport function resulted in decreased DHA transport and increased activity levels of transcytosis within CNS endothelial cells (Andreone et al., 2017). A leaky barrier increases the brain’s susceptibility to toxins and pathogens and homeostasis disruption, and ultimately, neuronal dysfunction (Abbott, 2000; Hutchinson, 2010; Ikeshima-Kataoka and Yasui, 2016; Block, 2019).

#### The Contribution of the BBB to AD Pathology

Loss of BBB function may contribute to neurodegenerative diseases, including AD (Banks, 1999; Gilgun-Sherki et al., 2001; Zlokovic, 2008; Carvey et al., 2009; Karamanos et al., 2014; Sweeney et al., 2018; Katt et al., 2019). According to multiple independent studies, BBB breakdown in AD is demonstrated by decreased integrity of BBB tight junctions, pericyte and endothelial degeneration, RBC extravasation, and brain capillary leakages (Zlokovic, 2008; Carvey et al., 2009; de Vries et al., 2012; Nelson et al., 2016; Sweeney et al., 2018). A buildup of blood proteins and macromolecules due to barrier leakiness may damage vasculature and brain parenchyma, which induces neuronal degeneration. Studies have also indicated that AD pathology includes reduced expression of glucose transporters in the BBB (Kalaria and Harik, 1989; Harik and Kalaria, 1991; Guo et al., 2005; Agrawal et al., 2017; Block, 2019). This may exacerbate AD cerebrovascular degeneration and cognitive function, considering that the brain requires a continuous supply of glucose and utilizes the most glucose of the major organs (Benton et al., 1996; Dienel et al., 1997; Benton, 2001; Gong et al., 2006). The BBB contains a wide variety of structural components to regulate the brain’s health and function, but a loss of function in any such component may lead to dyshomeostasis and a rapid cascade of dysfunctions in other structures within the brain.

### Lipids Contribute to Amyloid Precursor Protein Processing

#### Amyloid Precursor Protein Processing

Amyloid precursor protein (APP) is a type I transmembrane protein that is cleaved into amyloid β-peptide (Aβ) by β- and γ-secretases (Nunan and Small, 2000; Hartmann, 2012). APP is synthesized in the endoplasmic reticulum and is found in the highest concentrations in neuron’s *trans*-Golgi-network, suggesting that APP is associated with secretory pathways (Palacios et al., 1992; Stephens and Austen, 1996; Kitazume et al., 2001; Tam et al., 2014; Toh et al., 2017; Liu et al., 2019). There are two accepted proteolytic pathways for APP processing − non-amyloidogenic and amyloidogenic (Ishiura, 1991; Kojima and Omori, 1992; Sisodia, 1992; Roberts et al., 1994; Mills and Reiner, 1999; Soriano et al., 2001; Irizarry et al., 2004; Song et al., 2004; Chow et al., 2010; Wang et al., 2010; Tomita and Wong, 2011). The non-amyloidogenic pathway involves cleavage of APP by α-secretase at the plasma membrane, releasing soluble APPα (sAPPα) fragments into the extracellular environment, and normalizes AG genes and memory (Volmar et al., 2017). The amyloidogenic pathway involves cleavage of APP by β-secretase in early endosomes, releasing sAPPβ fragments in the endosomal lumen, and increasing susceptibility to Aβ plaques that are relevant to AD pathology (Estus et al., 1992; Golde et al., 1992; Saftig et al., 1996; Ehehalt et al., 2003; Andrew et al., 2016; Grimm et al., 2016).

#### The Role of Lipids in APP Processing

**FIGURE 2:**
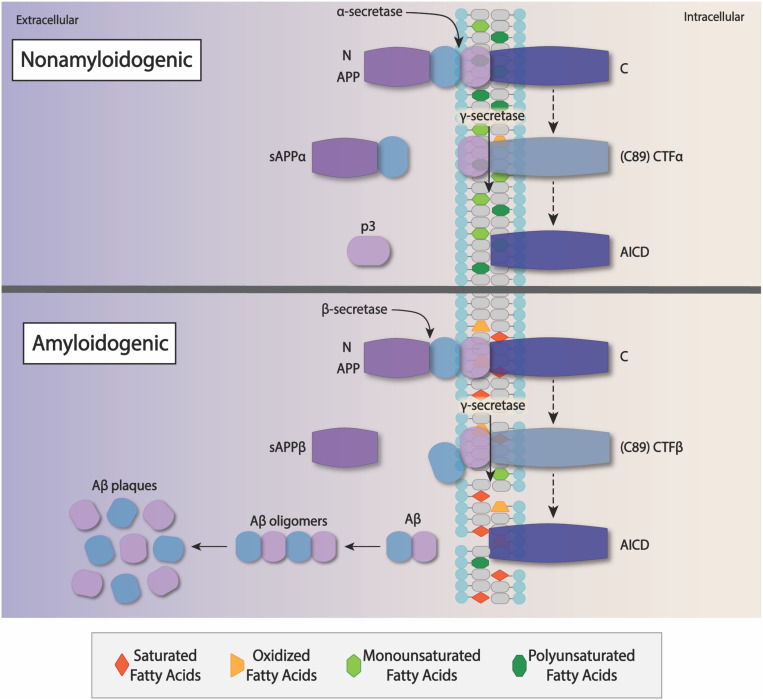
The importance of Lipids on APP processing – APP is a transmembrane protein that is cleaved by several proteases: α-secretase, β-secretases, and γ-secretases. *Non-amyloidogenic processing of APP*− In a cell with a membrane containing normal or high amounts of unsaturated fatty acids, especially DHA, preference is given to cleavage by α-secretase In this case, a well-structured membrane holds onto an intact APP as it is cleaved by the α-secretase and subsequently the α-secretase releasing the secreted ectodomain sAPPα, along with a small protein fragment, p3, and APP intracellular C-terminal domain (AICD) peptide in the extracellular space. sAPPα and p3 do not form neurotoxic fibrils and plaques, and so this process is referred to as non-amyloidogenic APP processing. *Amyloidogenic processing of APP* – In contrast, PUFA enriched structure of healthy neurons, the presence of saturated and oxidized fatty acids results in the disruption of the cell membrane structure, and this favors β-secretase activation. APP is cleaved at its’ N-terminus by β-secretase, releasing a soluble ectodomain sAPPβ into the extracellular space. γ-secretase subsequently cleaves the cell-associated C-terminus releasing and Aβ peptides of varying lengths into the extracellular space. Insoluble Aβ fibrils aggregate as oligomers that ultimately clump to form plaques within the brain. These plaques contribute to oxidative stress, neuroinflammation, and eventually decreased brain function.

The β-site APP-cleaving enzyme 1 (BACE-1) is the major β-secretase that targets endosomes with APP in transit to endocytosis sites on the plasma membrane (Shimokawa et al., 1993; O’Brien and Wong, 2011; Chun et al., 2015; Audagnotto et al., 2018). Both APP and BACE-1 are associated with lipid rafts, which are membrane domains enriched with cholesterol, sphingolipids, and gangliosides that are crucial to vesicle trafficking and intracellular transport (Ehehalt et al., 2003; Yoon et al., 2007; Marquer et al., 2011; Bhattacharyya et al., 2013). Recent studies have proposed that BACE-1 in cholesterol depleted environments displayed inhibited β-secretase activity, suggesting that cholesterol and lipid composition of the intracellular environment may be a large determinant of whether BACE-1 can access APP endosomes (Dash and Moore, 1993; Cheng et al., 2014; Mukadam et al., 2018). However, other studies suggest that both homeostasis of lipid composition and oxidation state of lipids, including DHA, are critical to APP processing (Grimm et al., 2012; Bhattacharyya et al., 2013; Figure 2). Under conditions with high concentrations of oxidized lipids, levels of sAPPα fragments decreased while sAPPβ levels increased (Grimm et al., 2016). A novel mechanism of proteolytic activity regulation of secretases involves a separating lipid boundary with their substrates, APP (Kaether and Haass, 2004). Lipid mediators of inflammation also interact with APP processing at the level of O-GlcNAcylation (Sastre et al., 2008; Jean-Louis et al., 2018). Thus, oxidized or inflammatory lipids may shift APP processing from the non-amyloidogenic to an amyloidogenic pathway (Figure 2).

#### The Intersection of Lipids, APP Processing, and AD Pathology

The Aβ fragments of APP is the major component of AD amyloid plaques, and such dysregulation of APP trafficking and processing are relevant to understanding AD pathology (Caporaso et al., 1994; Thinakaran and Koo, 2008; Zhang et al., 2011; Tan and Gleeson, 2019; Yuksel and Tacal, 2019). Intracellular Aβ accumulation in neurons of patients with AD and metabolic analysis of brain function indicate a possible dysfunction in Aβ transport exiting the brain (O’Brien and Wong, 2011; Yuksel and Tacal, 2019). Lipids rafts play important roles in APP trafficking (Yoon et al., 2007; Yang et al., 2013). Moreover, palmitoylation dictates how APP is processed (Bhattacharyya et al., 2013). *Trans* fatty acids influence amylogenic APP processing, while the level of fatty acid unsaturation determines the activity of secretases (Yang et al., 2011; Grimm et al., 2012). Future research relating to changes in brain lipid composition in pre-symptomatic AD may provide a link with early disease onset, dysregulation of lipid metabolism, and APP processing.

#### The Intersection of Lipid Rafts, APP Processing, and AD Pathology

Lipid rafts are dynamic clusters of membrane lipids that interact with protein complexes to promote intracellular signal transduction (Mesa-Herrera et al., 2019). Normal aging is associated with gradual reductions in cholesterol and polyunsaturated fatty acids (PUFAs) in lipid rafts. With age-related changes lipid rafts composition, alterations in intracellular communication may be associated with age-associated reductions in synaptic plasticity. In neurodegenerative diseases, the composition of lipid rafts changes more rapidly, most notably in n-3 and n-6 PUFAs (Li et al., 2018). Lipid raft aging appears to be exacerbated in Alzheimer’s Disease, which may serve as the underlying contribution to disrupted signal transduction, increased APP processing, and rapid formation of AB aggregates (Grassi et al., 2019). Normal APP signal transduction involves cleaving APP into AB into the extracellular environment. However, if APP interacts with ApoE and tau on a lipid raft with an atypical lipid composition, signal transduction may be disrupted, promoting the formation of AB aggregates. Other alterations include reductions in unsaturation of FA in AD patients, as compared to controls (Kao et al., 2020). Lipid raft aging also appears to exhibit gender differences, such that women had more severe changes in lipid raft composition as compared to men. This may serve as supportive evidence for the finding that postmenopausal women are more likely to progress from MCI to AD than age-matchd men (Herrera). Considering that lipid raft function is sensitive to aging, further characterization of composition changes in lipid rafts within the brain may be useful as a biomarker of neurodegenerative stages.

### Lipids and Cellular Remodeling

#### Role of Lipid Remodeling in Synaptogenesis

Lipid bodies (LBs) are spherical lipid-rich organelles associated with lipid storage, metabolism, cell signaling, and inflammation (Schmitz and Muller, 1991; Melo et al., 2011). At regulated levels, LBs maintain lipid homeostasis and cellular function, but in response to brain inflammation and increased neuronal oxidative stress, these LBs grow in size and accumulate within microglial cells (Tremblay et al., 2016; Hu et al., 2017). Though the pathway is still largely undiscovered, LBs in microglia appear to communicate with organelles such as the mitochondria, which control cell-death mechanisms (Tyurina et al., 2014). When exposed to lipopolysaccharides, LBs contact to mitochondria was disrupted, but DHA treatment reduced such effects. DHA may be a key factor in preserving mitochondrial health and regulation of microglial activity (Tremblay et al., 2016; Maysinger et al., 2018). When regulated in rodent models of AD, microglia slows the accumulation of Aβ plaques, but a proliferation of microglia activity may result in brain inflammation and degradation of neuronal synapses (Lim et al., 2000; Stahl et al., 2006; McClean et al., 2015). Microglial dysfunction has been implicated as a contributor to AD pathogenesis (Hansen et al., 2018). Microglia cells in the brain contribute to the reorganization of neuronal circuits by phagocytosing dead neurons and their dendritic spines and axon terminals. These immune cells contribute to neural plasticity (Wu and Zhuo, 2008; Yates, 2014; Yang et al., 2019), which refers to the brain’s ability to maintain, modify, and strengthen these synapses in order to permit neuronal communication (Tremblay et al., 2011).

#### Importance of Lipid Remodeling/Synaptogenesis in AD Pathology

Synaptogenesis is the formation of nerve synapses involving the reorganization of cell structural components (Aoki et al., 2003; Kelsch et al., 2010). Several studies suggest that presynaptic and postsynaptic development is initiated by signaling pathways involving cholesterol (Mauch et al., 2001; Fester et al., 2009). Changes in fatty acid content occur prior to synaptogenesis in cones (Martin and Bazan, 1992). Studies have shown that neurons deprived of lipid rafts underwent a cascade of effects inhibiting synaptic growth and development (Bazan, 2005; Welberg, 2014; Mochel, 2018). Depletion of lipid rafts decreased dendritic density and increased the synapse, disrupting neuronal communication (Martin, 2000; Hering et al., 2003; Sebastiao et al., 2013; Wang, 2014). The transport protein, apolipoprotein E (apoE), monitors cholesterol transport from glial cells to neurons, and impaired ApoE is implicated in deficits in synaptic plasticity and cognitive function (Periyasamy et al., 2017). Of the three isoforms of ApoE, ApoE4 is a prevalent risk factor that is synergistic with obesity and age for AD (Butler, 1994; Riedel et al., 2016; Jones and Rebeck, 2018; O’Donoghue et al., 2018; Glorioso et al., 2019). ApoE4 binds fewer lipids and is most likely involved in changes in cholesterol flux and metabolism (de Chaves and Narayanaswami, 2008; van den Kommer et al., 2012; Mahley, 2016; Nunes et al., 2018), accounting for altered synaptogenesis and neural plasticity.

### Lipids and Myelination

#### The Importance of Myelination

Action potentials propagate along axons through rapid saltatory conduction. Synthesized by oligodendrocytes in the CNS and Schwann glial cells in the PNS, myelin membranes act as electrical insulators, permitting higher nerve conduction velocities and greater neuronal communication efficiency (Almeida and Lyons, 2014; Almeida and Lyons, 2017). Without myelin, axons would require more energy to depolarize its membrane (Stassart et al., 2018). Myelin is composed of several lipids and protein layers that wrap around most of the axon, except at nodes of Ranvier, which are regions highly concentrated with sodium ion channels (Finean and Robertson, 1958; Davison, 1972; Burgisser et al., 1986; Wender et al., 1988; Ando et al., 2003; Schmitt et al., 2015; Montani and Suter, 2018). Myelination of axons is a dynamic process through development and adulthood, and this process, in addition to myelin sheath modification and myelin repair, contributes to synaptic remodeling and neural plasticity (Zatorre et al., 2012).

#### The Role of Lipids in Myelination

The myelin membrane consists of myelin-specific proteins and high-level synthesis of lipids representative of all major classes, such as cholesterol, glycosphingolipids, glycerophospholipids, and galactolipids (Chrast et al., 2011). Lipids comprise approximately 80% of myelin’s dry weight, accounting for glia’s high demand for fatty acids, which are fundamental building blocks of its lipid structure (Dimas et al., 2019). Myelin accounts for a majority of the white matter in the brain, which is consistent with reported reduced myelin density associated with AD white matter changes in the brain (Nasrabady et al., 2018).

### Brain Myelination and AD Pathology

Reduced number and activity of oligodendrocytes and precursor cells can damage myelin integrity, contributing to AD pathology’s characteristic neuronal loss (Bartzokis, 2011). Oligodendrocytes support and regulate neurons, but they are primarily responsible for myelin production (Simons and Nave, 2015). Myelinating oligodendrocytes are sensitive to lipid peroxidation because oxidative stress inhibits expression of genes that promote oligodendrocyte differentiation (French et al., 2009). This implies that disruption of myelin synthesis may be a central feature of AD pathology, and can be exploited for therapy (Desai et al., 2010). Dysfunction in these processes may be linked to white matter abnormalities and cognitive impairment associated with AD due to damaged signal conductivity and synchronicity needed for information processing between neurons (Ihara et al., 2010; Alexander, 2017; Nasrabady et al., 2018). The causal relationship between myelination and AD has not been elucidated, but white matter changes arising from myelination dysfunctions have been described in AD brains (Kohama et al., 2012). Additional evidence for the contribution of myelin breakdown on AD pathology comes from studies showing that the rate and severity of myelin breakdown in healthy seniors are associated with APOE status, a major risk factor of AD (Bartzokis et al., 2006).

### Lipids and Receptor-Mediated Signaling

#### Neuronal Receptor Signaling Pathways

Neurons communicate via electrochemical signals and neurotransmitters across gaps called synapses associated with several integrated networks (Mayer, 1993; Laughlin and Sejnowski, 2003; Salinas, 2009; Hahn et al., 2019). The presynaptic neuron releases neurotransmitters through exocytosis, and those chemicals bind to the postsynaptic neuron’s neurotransmitter receptors to alter postsynaptic neuronal activity (Kennedy, 2013). One class of neurotransmitter receptors, called ligand-gated ion channel receptors, opens an ion pore through the membrane upon ligand binding. Ions cannot travel through the hydrophobic lipid membrane and, therefore, can only pass through channels controlled by these receptors. Ions entering the ligand-gated channel can initiate excitatory or inhibitory signals, but both rapidly influence neuronal function (Cantor, 2018). Another class of neurotransmitter receptors, G-protein-coupled receptors (GPCRs), bind to the ligand and initiate an intracellular mechanism in which its G-proteins alter cAMP levels to stimulate or inhibit the neuron, and may involve lipid agonists (Hansen, 2015). Unlike ligand-gated ion channel receptors, GPCRs are slower but longer-lasting in affecting neuronal activity (Lovinger, 2008).

#### Role of Lipids in Neuronal Signaling

While cascades of protein kinases and phosphatases have been largely studied, there is an increasing interest in lipid-based pathways involving lipid kinases and phosphatases. Lipids are versatile in signal transduction pathways and act as hormones, ligands, substrates, and mediators (Eyster, 2007; Piomelli et al., 2007; Piomelli, 2012). Sphingolipids and cholesterol comprise lipid rafts, which are regions in the plasma membrane that organize signaling molecules, amplify intracellular signaling cascades, and regulate both neurotransmission and membrane protein trafficking (Levental and Veatch, 2016). Additionally, lipids are integral to GPCR signaling cascades. Following GPCR binding, phospholipase C (PLC) cleaves the polar phosphate head of phospholipids and forms diacylglycerol (DAG), a lipid second messenger (Black et al., 2016). Fatty acids (FAs), especially those belonging to the omega-3 and omega-6 classes, act as ligands for membrane receptors in a variety of pathways (Mobraten et al., 2013). The wide diversity of lipids and their structures contributes to AD, and their multiple roles in signal transduction may influence AD pathology.

#### Signaling Lipids Contribute to AD Pathology

Endocannabinoid signaling is responsible for inhibition and excitation in modulating synaptic strength, implicating its possible role in AD and associated inflammatory pathology (Skaper and Di Marzo, 2012). Although the mechanism has not been elucidated yet, free radicals and oxidative stress increase GPCR cannabinoid 2 receptors (CB2) expression in AD microglial cells, increasing neuroinflammation (Paloczi et al., 2018). Inflammation protects the brain against neurotoxins, but excessive inflammation may contribute to neurodegeneration. Another study suggested that monoacylglycerol lipase (MAGL) produces neuroinflammatory prostaglandins through the hydrolysis of endocannabinoids (Piro et al., 2012). Inhibiting MAGL activity is a potential AD therapeutic target because it is reported to prevent neuroinflammation, neurodegeneration, and impaired synaptic plasticity (Chen et al., 2012). Dysregulation in neuronal signaling cascades may contribute to increased susceptibility to neuronal dysfunction and are, therefore, important in studying its effects and relation to AD.

### Lipids and Inflammation

#### The Importance of Inflammation

Inflammation is a defense mechanism initiated by the immune system in response to pathogens, injured cells, infections, and other toxic stimuli. A signaling cascade results in leukocyte migration to damaged sites, in which released cytokines recruit other immune cells to heal injured tissue (Robinson et al., 2018). Specifically, within the CNS, activation of microglia and its associated cytokine production are primarily responsible for the inflammatory responses (Frank et al., 2007; Ghosh et al., 2012; Zhu et al., 2019). However, unregulated inflammation, excessive cytokine production, and failure to resolve inflammatory responses all contribute to chronic neuroinflammation, a biomarker of many neurodegenerative diseases, including AD (Wang et al., 2015a).

#### Lipids and Inflammation

Several studies implicate the role of lipids and lipid metabolism in inflammatory responses (Janciauskiene and Wright, 1998; Kang and Rivest, 2012; Zhang et al., 2018; Ntambi, 2019). Eicosanoids are a class of lipid mediators inflammation produced by innate immune cells that contribute to acute inflammation, resulting in pain, loss of function, heat, and swelling (Higgs et al., 1984; Williams and Higgs, 1988; Hedqvist et al., 1991; Umamaheswaran et al., 2018). Following the elimination of toxic stimuli, innate immune cells cease the production of eicosanoids and begin production of specialized pro-resolving lipid mediators (SPMs) to resolve inflammation (Serhan, 2010; Chandrasekharan and Sharma-Walia, 2015; Chiurchiu et al., 2018; Maclean et al., 2018). Synthesized from omega-3 fatty acids, docosahexaenoic acid (DHA), and eicosapentaenoic acid (EPA), SPMs resolve inflammation by inhibiting polymorphonuclear leukocytes (PMN) and lowering vascular permeability This process may be impaired in AD (Whittington et al., 2017).

#### Inflammatory Lipids and AD Pathology

A disproportionate level of inflammation can disrupt the balance between eicosanoids and SPMs, overwhelming the brain’s ability to return to a non-inflammatory state. This suggests the brain’s dependence on SPMs and its omega-3 precursors, DHA, and EPA (Serhan et al., 2018). AD pathology includes decreased DHA levels (Fonteh et al., 2014; Yassine et al., 2017), which may account for heightened brain inflammation that leads to declining cognitive health. Moreover, many studies have reported alterations to the eicosanoid pathway in AD (Biringer, 2019), further heightening research interest in the balance between eicosanoids and SPMs (Serhan et al., 2015). AD is also associated with elevated microglia-induced neuroinflammation, increases in proinflammatory cytokines, and upregulated expression of phagocytic receptors in white matter microglia (Zheng et al., 2016). One receptor, CD36, promotes both pro-inflammatory and oxidative pathways upon binding to ligands, including lipids and Aβ (Doens et al., 2017). Overexpression may lead to dysregulated inflammation and increased oxidative stress, a biomarker of the inflammatory response, and AD (Park et al., 2014; Koizumi et al., 2016). White matter is critical to neuronal connectivity and processing speed, and such white matter inflammation may result in neurodegeneration and, therefore, the cognitive decline (Raj et al., 2017). Further studies aim to determine if inflammation contributes to the onset of AD or exacerbates already-existing neuropathology (Heppner et al., 2015).

### Lipids and Oxidative Stress

#### Oxidative Stress

Oxidative stress is defined as a disruption in homeostasis between antioxidants and oxidants, and more specifically, an accumulation of reactive oxidative species (ROS) and reactive nitrogen species (RNS) (Apak et al., 2016; Hameister et al., 2020). ROS belongs to a family of compounds containing partially reduced oxygen species, such as O_2_– and HO-, that are generated primarily by the electron transport chain during aerobic respiration (Zhao et al., 2019). ROS are involved in many redox-dependent processes, including cell signaling, homeostasis, immune system responses, energy metabolism, and tissue remodeling. However, an excess of ROS or impaired control of the balance between antioxidants and oxidants leads to oxidative stress, which is implicated in the progression of neurodegenerative diseases (Cheignon et al., 2017). Because the brain consumes approximately 25% of the body’s glucose, its high energy consumption increases neurons’ susceptibility to oxidative stress and overproduction of ROS (Wezyk et al., 2018).

#### Membrane Lipids Are Damaged During Oxidative Stress

Excess ROS can lead to increased lipid peroxidation within the brain, altering membrane permeability and activity of membrane receptors and their associated enzymes (Birben et al., 2012). Lipid peroxidation produces reactive aldehydes, including malondialdehyde (MDA) and 4-hydroxynonenal (HNE), that modify and bind to proteins involved in metabolism, antioxidant defense systems, and axonal growth. By modifying Tau protein, 4-HNE can indirectly lead to an increase in neurofibrillary tangles, which is consistent with proteomic reports of increased 4-HNE in AD hippocampal tissue and neurofibrillary tangles (Cheignon et al., 2018). Moreover, low-density lipid lipoprotein receptor-related protein (LRP1) is involved in Aβ peptide removal. LRP1 is oxidized by Aβ, inhibiting its ability to clear Aβ and therefore leading to Aβ accumulation in the brain (Shinohara et al., 2017). LRP1 is another protein that is covalently modified by 4-HNE, further supporting that unrestrained lipid peroxidation produces excess reactive products that initiate a cascade of dysregulations within pathways necessary to neuronal health (Butterfield et al., 2002). Oxidant/antioxidant imbalance forms blood-based biomarkers that can be used for early, non-invasive diagnosis (Wojsiat et al., 2018), or for AD therapies (Yatin et al., 2000; Sultana et al., 2004).

#### Oxidative Stress and AD Pathology

Many trials seek to assess different antioxidant therapeutic approaches to alleviate oxidative stress, a key biomarker of AD. CoQ_10_, creatine, idebenone, latrepirdine, triterpenoids, omega-3 PUFAs, vitamin E, and vitamin C are just a few antioxidants that are extensively studied in their treatment of neurodegenerative diseases (Yatin et al., 2000; Kumar and Singh, 2015).

### Lipids and Immune Response

#### The Immune System

The immune system, which is divided into the innate and adaptive immune system, is critical to defending the body against infectious and toxic stimuli (Simon et al., 2015). The innate immune system utilizes cytokine production and modulation to mount a quick but sufficient response to pathogens, including viruses, bacteria, and parasites. The innate immune system is also responsible for activating the adaptive immune system, which is slower due to the lengthy production of specific antibodies to the foreign antigen (Iwasaki and Medzhitov, 2015). Studies in the past 20 years have refuted the notion of the brain as being “immunologically privileged” in relying largely on innate immune system mechanisms within the CNS. While it was thought that the CNS and immune system were separate due to the blood-brain barrier, the detection of lymphatic vessels connecting T-cells in lymph nodes to cerebrospinal fluid (CSF) in the meninges provided evidence for the brain’s semi-dependence on the adaptive immune system (Louveau et al., 2015). Neuroimmune processes are activated by vagal nerve signaling, immune signals, and complement proteins, resulting in increased activity of microglia and astrocytes (Tchessalova et al., 2018).

#### Lipids and Immunity

Studies reported increased levels of platelets and vascular lesions in AD patients outside of the brain, contributing to cerebral amyloid angiopathy, a biomarker of AD that shows increased amyloid protein in the brain arteries (Kniewallner et al., 2015). Although platelets combat vascular injury, they are also involved in APP processing, and transitively, the formation of Aβ plaques (Evin et al., 2003; Evin and Li, 2012). The balance of omega-3 and omega-6 PUFAs may affect platelet levels, as membrane essential fatty acids (EFAs), primarily DHA and EPA, form prostaglandins PGE_1_, PGE_2_, and PGE_3_, all of which participate in a variety of immunological and signaling pathways in the brain (Chang et al., 2009). PGE_1_ has anti-inflammatory properties, and conversely, PGE_2_ strongly promotes inflammation by acting on different receptors (Iyu et al., 2011). PGE_3_ is responsible for regulating PGE_2_’s inflammatory effects by competing with its formation from precursor EFAs (Chang et al., 2009). Imbalances in the omega-6 to omega-3 PUFA ratios disrupt the formation of PGE_3_, which minimizes the regulation of PGE_2_ induced inflammation. Moreover, this imbalance of PUFAs is associated with changes in neuronal brain composition that, in combination with drug therapies, can reduce the risks and slow the progression of AD (Giulietti et al., 2016).

#### Immunity and AD Pathology

An impaired BBB is implicated with the onset of AD, which may increase the BBB’s permeability to pathogens and immune cells (Veerhuis et al., 2011). Levels of cytotoxic and helper T-cells are upregulated in brain parenchyma of AD patients (Oberstein et al., 2018). Helper T cells and pro-inflammatory cytokines target neurofibrillary tangles and plaques composed of Aβ and Tau and activate microglia at these sites, further exacerbating neuroinflammation (Gold et al., 2014; Martinez-Frailes et al., 2019). One class of cytokines, called chemokines, stimulates leukocyte migration from blood to tissues. CCL5 is a chemokine that is amplified in response to reactive oxygen species and oxidative stress within the brain’s endothelial cells, promoting even more T cell migration across the leaky BBB. These inflammatory mediators are elevated in the CSF and blood and are possible biomarkers for detecting AD and its progression (Mietelska-Porowska and Wojda, 2017).

### Lipids and Energy Regulation

#### Sources of Brain Energy

Although the human brain comprises only 2% of the body weight, it consumes approximately 20% of glucose, demonstrating its disproportionately high energy demand (Mergenthaler et al., 2013). The majority of the energy utilized by the brain is dedicated to returning neurons to their resting states after depolarization, and the remaining 20−25% of energy is allocated toward synthesizing vesicles and neurotransmitters (Harris et al., 2012). The brain relies on a constant flow of glucose and oxygen, which are delivered through the blood. However, during fasting periods, when glucose levels are decreased, the liver can supply ketone bodies to support metabolism within the brain (Patel et al., 1975; Hawkins and Biebuyck, 1979; Nehlig, 2004). These delivered ketone bodies are primarily utilized by astrocytes, and upon arrival, ketolysis of the ketone bodies generates acetyl CoA, an important substrate for the tricarboxylic acid (TCA) cycle and therefore, ATP production. Although the brain has a large ATP requirement, it does not utilize these ketone bodies or fatty acids as a significant source of energy like in other organs, such as the liver. It is hypothesized that evolution selected against this pathway because it produces ROS and therefore, contributes to oxidative stress that contributes to neurodegeneration (Schonfeld and Reiser, 2017).

#### Role of Brain Energy Regulation in AD Pathology

Transport and utilization of glucose within the brain are tightly regulated, but mitochondrial dysfunction and decreased expression of glucose transporters (GLUT) are potential contributors to AD (Yin et al., 2016). Highly concentrated in the BBB, GLUT1 transports glucose across the endothelium and into astrocytes, whereas GLUT3 is predominantly found in axons and dendrites, underscoring its role in neuronal glucose transport and distribution (Vannucci et al., 1998). Reduced GLUTs expression at the BBB and within neurons is associated with AD, which may explain overall decreased glucose metabolism in AD pathology (Yin et al., 2016).

#### Mitochondrial Dysfunction and AD Pathology

Mitochondria are organelles central to brain energy processes, and altering glucose availability or dysregulating oxidative phosphorylation can have direct effects on neuronal function and cognitive health (Picard and McEwen, 2014; Anderson, 2018). Recent reports have hypothesized that Aβ may initiate mitochondrial dysfunction, and one theory proposes that Aβ raises cytosolic calcium levels, inhibiting oxidative phosphorylation and, therefore, ATP production (Cardoso et al., 2001; Eckert et al., 2010; Spuch et al., 2012; Kaminsky et al., 2015; Brewer et al., 2020). Moreover, mitochondria delivery to needed brain regions is dependent on tau, a protein related to microtubules (Quintanilla et al., 2012; Amadoro et al., 2014). Mitochondria are observed to be differentially localized in AD brains, suggesting that mitochondria trafficking is affected (Nicholls and Budd, 2000; Duchen, 2012; Devine and Kittler, 2018; Son and Han, 2018; Rangaraju et al., 2019), and provides further support for mitochondrial-based contributors to neurodegeneration.

## Potential AD Therapies Targeting Lipid Metabolism

### Dietary Modification Studies

With the realization that lipids are altered in AD pathology, several studies have identified specific lipids that may be used as dietary supplements to alleviate AD symptoms (Table 3). The major lipids include omega-3 fatty acids (DHA, EPA), choline-containing lipids, cholesterol, and lipids with antioxidant properties (CoQ_10_, Vitamin K).

**TABLE 3:** Lipid diets and their effects on AD.

Lipid diet interventions	Effects on AD
Algal DHA^#^; 2 g daily for 18 months	Supplementation with DHA compared with placebo did not slow the rate of cognitive and functional decline in patients with mild to moderate Alzheimer’s disease (Quinn et al., 2010).
Consumption of fish once or more per week	In adults above the age of 65, participants who consumed fish once or more per week had 60% less risk of developing Alzheimer’s compared to participants who rarely or never ate fish (Morris et al., 2003).
Omega-3 PUFA; 600 mg EPA and 625 mg DHA daily for 4 months	In adults with mild cognitive impairment and probable AD, omega-3 supplementation had negligible effects on cognition or mood (Phillips et al., 2015).
EPA-DHA for 26 weeks; stratified into high-dose (180 mg EPA-DHA daily) and low-dose (400 mg daily)	In cognitively healthy adults over 65 years old, there were no significant differential changes in any of the cognitive domains for either low-dose or high-dose fish oil supplementation compared with placebo (van de Rest et al., 2008).
Study participants are postmenopausal women (60−84 years); 1g DHA, 160 mg EPA, 240 mg Ginkgo biloba, 60 mg PS, 20 mg per day for 6 months	In a randomized, double-blind study, a high dose of omega-3 nutrients has cognition and mobility benefits to older women (Strike et al., 2016).
DHA-EPA; 1.7 g DHA and 0.6 g EPA daily for 6 months (OmegAD Study)	Omega-3 fatty acids did not delay the rate of cognitive decline, nor did it have marked effects on neuropsychiatric symptoms except for possible positive effects on depressive symptoms in non-APOE4 carriers and agitation symptoms in APOE4 carriers (Freund-Levi et al., 2006; Freund-Levi et al., 2008). Plasma levels of AA decreased while DHA and EPA levels increased at 6 months. Specialized pro-resolving mediators (SPMs) do not change in the omega-3 group but a decrease in the placebo group. SPM changes associate with cognitive changes in AD (Lopez et al., 2011).
Omega-3 PUFAs; 1.8 g daily for 24 weeks	The omega-3 supplementation treatment group showed significant improvement in the Alzheimer’s Disease Assessment Scale compared to the placebo group in participants with mild cognitive impairment. However, there was no significant improvement in Alzheimer’s disease study participants (Chiu et al., 2008).
Supplementation with omega-3 fatty acids alone or omega-3 plus alpha-lipoic acid; 675 mg DHA and 975 mg EPA or 675 mg DHA and 975 mg EPA plus 600 mg lipoic acid daily for 12 months	Combining omega-3 fatty acids with lipoic acid slowed both cognitive and functional decline in mild to moderately impaired AD participants over 12 months compared to placebo (Shinto et al., 2014).
3 DHA exposure variables used in separate analyses; plasma DHA, dietary DHA, and consumption of cold-water fish	Plasma and dietary DHA were associated with a decreased risk of dementia and AD (Lopez et al., 2011).
Arachidonic acid and DHA supplementation;240 mg of AA and DHA daily for 90 days	Participants with mild cognitive impairment showed a significant improvement in the immediate memory and attention score compared to placebo, but there was no significant improvement in participants with AD (Kotani et al., 2006).
Docosahexaenoic acid-concentrated fish oil supplementation; 430 mg of DHA and 150 mg of EPA daily for 12 months	In participants with mild cognitive impairment, supplementation resulted in a significant improvement in short-term memory, working memory, immediate verbal memory, and delayed recall capability (Lee et al., 2013).
Fortasyn Connect supplementation; 125 mL once-a-day drink containing Fortasyn Connect for 24 months (LipiDiDiet Trial)	In individuals with prodromal AD, Fortasyn Connect supplementation had no significant effect on neuropsychological test battery results (Soininen et al., 2017).
FINGER Study − Dietary intervention using a diet with 10−20% of daily energy (E%) from proteins, 25−35% from fat (less than 10E% from SAFA, 10−20% from MUFA, 5−10% from PUFA (including 2,5−3 g/day n-3 fatty acids); 45−55% from carbohydrates (less than 10% refined sugar); 25−35 g/day dietary fiber; less than 5 g/day salt; and less than 5 E% from alcohol for 2 years	In adults over 60 years old, there was a significant beneficial intervention effect on overall cognitive performance, including memory, executive function, and psychomotor speed (Rosenberg et al., 2020).

*^#^DHA, Docosahexaenoic acid (C22:6, n-3); EPA, Eicosapentaenoic acid (C20:5, n-3); MUFA, monounsaturated fatty acids; PUFA, polyunsaturated fatty acids; SAFA, saturated fatty acids.*

Several dietary intervention studies using DHA have yielded mixed effects on AD symptoms. A likely reason for these mixed results is that different disease severity, different formulations, and variable endpoint and time of interventions were studied (Fonteh, 2018). Recent studies indicate that the best form of DHA delivery into the brain is through the Msf2a LPC receptors (Sugasini et al., 2019). A better understanding of the right formulation and optimal concentrations of DHA probably supplemented at the prodromal phase of AD will likely yield beneficial outcomes.

### Modification of Lipid Metabolism

Metabolism of lipids can be altered to prevent depletion of their levels in the AD by targeting pathways that transport or catabolize these lipids in the brain.

### Lipid Transport Into the Brain

Several lipoproteins and their receptor complexes are the major form by which lipids bypass the BBB to be delivered into the brain. Several of these lipoprotein genes are linked to AD pathology (Table 1). Some lipoproteins have protective effects, while others have AD enhancing properties. For example, HDL has been shown to be protective by improving Aβ clearance, delaying Aβ fibrillization, suppressing vascular inflammation, and inducing endothelial nitric oxide production (Button et al., 2019).

### Cholesterol Metabolism

Since cholesterol metabolism altered at several stages of AD, modulation of its metabolism may have beneficial effects on disease progression. Modification of cholesterol homeostasis can be influenced during its consumption, at the level of its biosynthesis, and during its transport into the brain. The use of statin to alter cholesterol biosynthesis is proposed to be insightful in AD pathophysiology and therapy (Wolozin et al., 2004; Hoglund et al., 2005; Biondi, 2007; Evans et al., 2009). Gene therapy targeting cholesterol 24-hydroxylase reduces the amyloid pathology before or after the onset of amyloid plaques in mouse AD models (Hudry et al., 2010). Studies in mouse models show that blocking the conversion of cholesterol to cholesterol esters has beneficial effects on AD (Shibuya et al., 2015). The relationship between hypercholesterolemia, cholesterol-lowering therapies, and the role of oxysterols in AD pathology have led to the proposition that cholesterol metabolites are valuable targets for alternative AD treatments or prevention (Loera-Valencia et al., 2019). Neuroinflammatory pathways mediated by toll-like receptor 4 (TLR4)-mediated signaling can aggravate AD symptoms. In a rodent AD model, treatment with an anti-inflammatory steroid (atorvastatin) regulates this inflammatory process and results in the amelioration of cognitive deficits (Wang et al., 2018).

### Lipolytic Enzymes

The activity or expression of several lipolytic enzymes are altered in AD. Phospholipase A_2_ (PLA_2_) is associated with amyloid plaques, and reduction of its activity and expression ameliorates AD. Plasmalogen selective PLA_2_ is also altered in AD. Our studies show an increase in PLA_2_ activity of CSF of AD participants accompanied by an increase in lysophosphatidylcholine (LPC). LPC is known to disrupt the BBB, and changes in PLA_2_ are associated with inflammation. The association of PLA_2_ with AD pathology suggests that inhibitors of PLA_2_ activity or expression may be an effective means of preventing AD. Ong et al. (2015) reviewed the importance of several natural and synthetic PLA_2_ inhibitors on the treatment of neurological disorders. Since PLA_2_ isoforms may have divergent effects on membrane remodeling and function, there is a need for isoform-specific inhibitors in order to avoid toxicity encountered with non-selective inhibitors. In addition to PLA_2_, phospholipase D (PLD) and phospholipase C (PLC) expression and activities are associated with AD pathology. These lipases that are linked with neurite growth and signaling, respectively, offer other avenues for exploring AD treatments.

### Lipid Oxidation Inhibitors

There is convincing evidence for the importance of oxidative stress on AD pathology (Sun et al., 2001; Bassett and Montine, 2003; Bacchetti et al., 2015). The most important brain fatty acid, DHA, is a polyunsaturated fatty that is easily susceptible to oxidative damage. While HDL is protective against oxidative damage, VLDL is easily oxidized. Oxidatively damaged lipids contribute to AD pathology by their highly neurotoxic properties (Bassett et al., 1999). Approaches that reduce oxidation are expected to reduce AD progression. These include the use of natural antioxidants, carnosine, lipoic acid, Ginkgo biloba flavonoids, soybean isoflavones, vitamin K, homocysteine, curcumin (Rutten et al., 2002; Vina et al., 2004; Frank and Gupta, 2005; Mancuso et al., 2007; Zhao, 2009; Cankurtaran et al., 2013). A limitation of natural antioxidant is the lack of demonstration of efficacy. Given that oxidative stress destroys mitochondrial function, an objective measure of any antioxidant can be their ability to restore mitochondrial function (Kumar and Singh, 2015; Kwon et al., 2016; Yu et al., 2016). The role of endogenous lipids in oxidative stress can be exploited when there is an uncontrolled formation of ROS and RNS or when the antioxidants contribute to disease pathology (Leuti et al., 2019). Also, the source of ROS determines the effects on cellular physiology and manipulation of the ubiquinone redox state is proposed to be a viable approach of delaying aging and therapy (Scialo et al., 2016; Wojsiat et al., 2018).

## Concluding Remarks

Biochemical, physiological, and genetic analyses show that lipid metabolism interphases with all the major facets of AD pathology (Figure 1). In normal aging, lipid metabolic homeostasis ensures that the basic functions of the brain are met. In AD, there is dyshomeostasis of lipid metabolism, and this results in abnormal functions of the brain that characterize disease progression. This underscores the need for detailed analyses of brain lipid homeostasis in order to unravel more comprehensive mechanisms, specific biomarkers, and novel therapies of AD.

## Author Contributions

AF contributed to the conceptualization and study design, supervised the data, and carried out the project administration and funding acquisition. HC, VS, and AF contributed to writing of the original draft and manuscript preparation. HC and AF contributed to the manuscript review and editing. VS and AF prepared the figures and illustrations.

## Conflict of Interest

The authors declare that the research was conducted in the absence of any commercial or financial relationships that could be construed as a potential conflict of interest.
